# Lateralized Deficits of Disgust Processing After Insula-Basal Ganglia Damage

**DOI:** 10.3389/fpsyg.2020.01429

**Published:** 2020-06-30

**Authors:** Olga Holtmann, Maximilian Bruchmann, Constanze Mönig, Wolfram Schwindt, Nico Melzer, Wolfgang H. R. Miltner, Thomas Straube

**Affiliations:** ^1^Institute of Medical Psychology and Systems Neuroscience, University of Muenster, Muenster, Germany; ^2^Otto Creutzfeldt Center for Cognitive and Behavioral Neuroscience, University of Muenster, Muenster, Germany; ^3^Department of Neurology, University Hospital Muenster, Muenster, Germany; ^4^Institute of Clinical Radiology, University Hospital Muenster, Muenster, Germany; ^5^Department of Clinical Psychology, Friedrich Schiller University Jena, Jena, Germany

**Keywords:** insula-basal ganglia system, disgust, hemispheric lateralization, lesion analysis, structure-function relationship

## Abstract

A growing body of evidence suggests a role of the insular cortex (IC) and the basal ganglia (BG) in the experience, expression, and recognition of disgust. However, human lesion research, probing this structure-function link, has yielded rather disparate findings in single cases of unilateral and bilateral damage to these areas. Comparative group approaches are needed to elucidate whether disgust-related deficits specifically follow damage to the IC-BG system, or whether there might be a differential hemispheric contribution to disgust processing. We examined emotional processing by means of a comprehensive emotional test battery in four patients with left- and four patients with right-hemispheric lesions to the IC-BG system as well as in 19 healthy controls. While single tests did not provide clear-cut separations of patient groups, composite scores indicated selective group effects for disgust. Importantly, left-lesioned patients presented attenuated disgust composites, while right-lesioned patients showed increased disgust composites, as compared to each other and controls. These findings propose a left-hemispheric basis of disgust, potentially due to asymmetrical representations of autonomic information in the human forebrain. The present study provides the first behavioral evidence of hemispheric lateralization of a specific emotion in the human brain, and contributes to neurobiological models of disgust.

## Introduction

The insular cortex (IC) and the basal ganglia (BG) have been attributed a pivotal role in the functional neurocircuitry of disgust (Chapman and Anderson, 2012). A substantial body of functional neuroimaging research has established that both regions are engaged in disgust experience (Wicker et al., 2003; Jabbi et al., 2008), disgust imagination [i.e., seeing (Wright et al., 2004; Calder et al., 2007), recollecting (Fitzgerald et al., 2004), and imagining (Jabbi et al., 2008) disgusting scenarios], and disgust perception in others (Phillips et al., 1998; Wicker et al., 2003; Jabbi et al., 2008]. Disproportionate deficits in recognition and experience of disgust are frequently seen in neuropsychiatric samples suffering from IC and BG dysfunction (e.g., Huntington’s disease, Wilson’s disease, Parkinson’s disease, or frontotemporal lobar degeneration) (for review see Vicario et al., 2017), and some studies unveiled associations between IC and BG atrophy and deficient disgust processing by means of voxel-based morphometry techniques (Kipps et al., 2007; Kumfor et al., 2013; Woolley et al., 2015; Verstaen et al., 2016). Epilepsy patients who received electrical stimulation in the anterior IC reported strong visceral sensations (e.g., nausea) that coincide with disgust (Mazzola et al., 2017). Another study on epilepsy patients revealed selective activation of depth electrodes in the anterior IC for disgusted rather than for other emotional faces (Krolak-Salmon et al., 2003).

While these lines of evidence strongly support the hypothesis of an IC-BG system in disgust, findings from studies of patients with focal lesions in the IC-BG complex are rather inconclusive. In a pioneering case study, selective impairment of recognition and experience of disgust was found in a patient with left-hemispheric infarction impacting both IC and BG (Calder et al., 2000). Another patient with bilateral IC, temporal and frontal damage exhibited disproportionate disgust deficits for dynamic emotional facial expressions and stories describing emotional actions (Adolphs et al., 2003). Several disgust-related deficits, besides self-disclosed reduction of overall intensity of emotional experience, were furthermore reported in a patient with damage to the left posterior IC (Borg et al., 2013). However, studies on patients with right-hemispheric damage failed to replicate these findings. No impairment of recognition or experience of disgust was found in a case of right-sided IC-BG infarction (Straube et al., 2010), although the extent of brain damage and the methodology applied were similar to the study by Calder et al. (2000). No deficits were reported in a multimodal assessment of emotional processing in a case of isolated IC damage (Couto et al., 2013). In contrast, deficient recognition of negative emotions from facial expressions was observed in three patients who were all suffering from right-hemispheric IC, frontal and temporal infarction (Terasawa et al., 2015). Deficits in multimodal recognition of aversive emotions were further reported in a patient with focal right-hemispheric damage of white matter association tracts adjacent to the IC (Couto et al., 2013). A recent multiple case study reported no substantial emotional deficits after unilateral operculo-insular resections in epilepsy surgery (Boucher et al., 2015). Studies on patients with focal brain lesions provide a crucial methodological approach to specify the necessity of brain regions attributed to them (Hillis, 2014). Thus, the inconsistency of lesion findings might point to a less decisive involvement of IC and BG in disgust processing. Another plausible option is distinct hemispheric involvement in disgust processing.

Influential models posit physiological body states as the basis for emotion and feeling (Craig, 2005; Critchley, 2005; Gu et al., 2013; Seth, 2013). The IC, along with the anterior cingulate (ACC), has been established as a cortical autonomic control center (Craig, 2005; Critchley, 2005). Cortical autonomic representations have been argued to be lateralized, with parasympathetic representations in the left, and sympathetic representations in the right hemisphere (Craig, 2005; Guo et al., 2016). Correspondingly, based on the autonomic representation, lateralized affective processing in the human forebrain has been suggested (Craig, 2005; Duerden et al., 2013; Kann et al., 2016). However, no consensus has been established on the exact nature of affective asymmetries (Duerden et al., 2013; Kann et al., 2016; Zhang et al., 2017). Distinct hemispheric involvement in disgust processing has been a matter of debate (Chapman and Anderson, 2012; Vicario et al., 2017). A large body of functional neuroimaging evidence suggests stronger left IC/BG activations to disgusting stimuli (Phillips et al., 1997; Zald and Pardo, 2000; Royet et al., 2003; Small et al., 2003; Wicker et al., 2003; Hennenlotter et al., 2004; Martin, 2013), but right (Phillips et al., 1997; Heining et al., 2003; Fusar-Poli et al., 2009) or bilateral (Phillips et al., 1997; Jabbi et al., 2008; Vytal and Hamann, 2010) activations are known as well. Left IC/BG atrophy has been linked to impaired disgust recognition in pre-symptomatic Huntington’s disease patients (Kipps et al., 2007; Henley et al., 2008) and frontotemporal dementia patients (Kumfor et al., 2013), but contrasting evidence suggests rather bilateral (Woolley et al., 2015) or right-sided associations (Adolphs et al., 2003). Crucially, direct electrical stimulation of the left IC in humans has been shown to cause a selective decrease of disgust recognition, as compared to other emotional facial expressions (Papagno et al., 2016). Electrical stimulation of the left IC in macaques has led to typical disgust grimaces (e.g., wrinkling of nose), retching and refuse of food intake (Caruana et al., 2011). However, lacking explicit comparative designs, previous methods of investigation have been limited to provide compelling data to support the hypothesis of a left-hemispheric basis of disgust. Systematic comparisons of patients with right- and left-lateralized brain damage matched for size and distribution are needed to clarify potentially distinct hemispheric contributions to disgust.

Disgust is a complex and multi-faceted emotion (Chapman and Anderson, 2012; Vicario et al., 2017). Although the IC-BG system is considered as a “multimodal disgust processor” (Calder et al., 2000; Wicker et al., 2003; Jabbi et al., 2008; Chapman and Anderson, 2012; Vicario et al., 2017), specific domains seem to be differently affected by IC-BG damage (Vicario et al., 2017). However, it is unclear which domain particularly draws on IC-BG functions. There is evidence of marked interindividual variability in disgust responsivity (Boucher et al., 2015; Tybur et al., 2018), as well as low consistency in the individual patterns observed across disgust domains (Woolley et al., 2015; Hall et al., 2016) in clinical and healthy samples. Thus, exploration of functional laterality by means of single methods appears rather limited (Duerden et al., 2013). These findings suggest that multiple measures are necessary to capture an adequate picture of disgust (Hall et al., 2016). In particular, indicators employing different stimuli and response domains may contribute to a broad assessment of dispositional disgust processing (Epstein, 1983). Aggregation across different measures has been suggested as a useful approach to address the depth and breadth of individual differences (Deater-Deckard et al., 2016), and to provide more robust dispositional estimates than single-test data (Epstein, 1983; Diener and Larsen, 1984; O’Neill et al., 2014). Thus, an integrative approach may provide conclusive evidence for the association between disgust and the IC-BG system, but has so far not been considered in patient-based research on disgust.

The primary aim of the present study was to systematically explore effects of lateralized damage to the IC and BG on disgust processing, in contrast to other basic emotions. We employed a comprehensive test battery addressing five basic emotional categories (disgust, anger, happiness, sadness, and fear) by means of well-established paradigms of emotion recognition and induction as well as questionnaires on the intensity and frequency of emotional experience. To better grasp dispositional emotional processing, we computed emotion-specific composite scores that combined information on processing each basic emotion from single methods. We examined four male patients with left and four male patients with right IC and BG damage who were matched on demographic and clinical parameters. Nineteen males with no history of psychiatric or neurological conditions served as healthy controls. In line with previous evidence (Woolley et al., 2015; Hall et al., 2016; Tybur et al., 2018), we expected heterogeneous task performance in patients and controls and, thus, a mixed pattern of findings at the single-test level. We assumed we would find selectively altered composite scores for disgust in the patients, when compared to controls, as would be consistent with the key role of the IC and BG in the functional neurocircuitry of disgust. We hypothesized that left, but not right, IC-BG damage would produce substantially reduced disgust composite scores.

## Materials and Methods

### Participants

**FIGURE 1:**
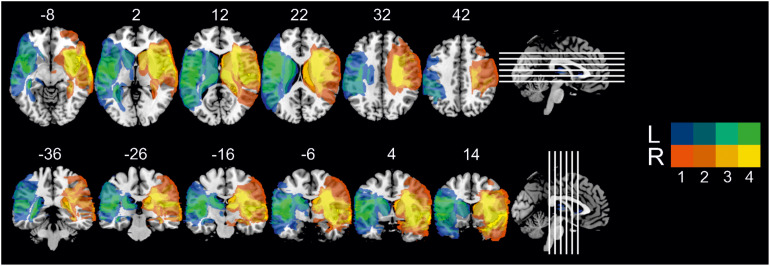
Overlay map of individual lesions determined in both patient groups, superimposed on a 1 mm MNI template, with MNI coordinates of each axial (z-axis, upper panel) and coronal section (y-axis, lower panel) provided for lesion localization. Color bars designate the number of patients with a lesion in this voxel (L = left-lesioned group, R = right-lesioned group).

Patients were recruited following participation in a rehabilitative program run by the Department of Clinical Psychology at the Friedrich Schiller University, Jena (Miltner et al., 1999, 2016). Inclusion criteria were: (1) unilateral lesions due to an ischaemic or haemorrhagic middle cerebral artery stroke, centrally affecting the IC-BG complex, as corroborated by high-resolution structural MRI data and neuroradiological report; (2) stable lesions (at least 1 year after lesion onset); (3) no cognitive deficits compromising understanding of instructions and task performance (i.e., global aphasia, attention deficits, amnesia, disorders of reasoning, visual neglect); (4) no history of neurodegenerative disorders, epilepsy, brain tumors, or brain trauma; (5) no history of substance induced disorders; (6) no history of psychiatric disorders; (7) motor ability to participate in the experimental procedure; (8) male gender, since previous single case findings on disgust processing have been mainly derived from the examination of male patients. Using these stringent inclusion criteria, eight patients with either left (*n* = 4) or right-hemispheric (*n* = 4) IC-BG damage due to ischemic (*n* = 7) or haemorrhagic stroke (*n* = 1) were found eligible and agreed to participate in the study. Single case and group-level demographical and clinical data are listed in Table 1. All patients were adequate in speech comprehension and speech production. Motor functions were sufficiently recovered to allow independent lifestyle. One patient (L3) suffered from diabetes type II. The left- and right-lesioned groups did not differ in terms of education ([*M* ± *SD*] 17.5 ± 1 years [left] vs. 15.5 ± 2.9 years [right], *t*_3_._71_ = 1.309, *P* = 0.266), age at testing (62.0 ± 12.4 years [left] vs. 55.5 ± 14.4 years [right], *t*_6_ = 0.685, *P* = 0.519), or age at diagnosis (53.5 ± 15.0 years [left] vs. 45.3 ± 17.9 years [right], *t*_6_ = 0.707, *P* = 0.506), resp. lesion age (8.5 ± 3.7 years [left] vs. 10.3 ± 4.3 years [right], *t*_6_ = 0.620, *P* = 0.558). On average, total lesion size (141.2 ± 22.8 cm^3^ [left] vs. 183.0 ± 50.9 cm^3^ [right], *t*_6_ = 1.503, *P* = 0.184) and anatomical distribution of damaged tissue were similar between the patient groups (see Figure 1 for the overlap of reconstructed lesions, Supplementary Figure 1 for individual anatomical images, Supplementary Table 1 for detailed whole-brain lesion analysis, and Supplementary Table 2 for detailed IC lesion analysis).

**TABLE 1:** Demographical and neuropsychological characteristics of patients (subject and group-level).

Patient	Laterality	Cause	Age at testing (years)	Age at diagnosis (years)	Lesion age (years)	Education (years)	Lesion size (cm^3^)	SIMD	Comorbi- dities	IQ (MWT-B, IQ)	Perceptual reasoning (WIE-BE, *z*)	Phasic alertness (TAP-Alert, *z*)	Selective attention (TAP-Go/NoGo, *z*)	Visual field/Neglect (TAP-VFCT, blind spots)	Visuo-spatial memory (CFT-CQM, *z*)	BDI-II
R1	Right	I	63	51	12	18	165	–	–	134	0	2.3	−2.2^a^	0	-0.7	2
R2	Right	I	64	59	5	13	187	–	–	108	1.67	0.3	−1.5	0	−0.1	16^b^
R3	Right	H	61	52	9	18	130	–	–	88	0	−1.7	−0.9	0	0.6	1
R4	Right	I	34	19	15	13	251	–	–	94	3.3	0.31	0.3	0	0.7	12
L1	Left	I	59	52	7	16	127	–	–	115	1	−0.8	−0.2	0	0.8	4
L2	Left	I	69	58	11	18	145	–	–	128	0	−1.4	0.3	0	1.2	4
L3	Left	I	74	70	4	18	120	–	diabetes type II	139	1	0.5	0.3	0	0.9	9
L4	Left	I	46	34	12	18	172	–	–	93	0	−0.6	−0.2	0	1.8	3
Right-lesioned group, mean (*SD*)			55.5 (14.4)	45.3 (17.9)	10.3 (4.3)	15.5 (2.9)	183.0 (50.9)	–	–	106.0 (10.5)	1.2 (1.6)	0.3 (1.6)	−1.1 (1.1)	0	0.1 (0.7)	7.8 (7.4)
Left-lesioned group, mean (*SD*)			62.0 (12.4)	53.5 (15.0)	8.5 (3.7)	17.5 (1.0)	141.2 (22.8)	–	–	118.8 (19.8)	0.5 (0.6)	−0.6 (0.8)	0.1 (0.3)	0	1.2 (0.5)	5.0 (2.7)
*P*-value			0.519	0.506	0.558	0.266	0.184	–	–	0.405	0.412	0.371	0.086	–	0.038*	0.526

BDI-II = Beck Depression Inventory, CFT-CQM = memory quotient from the Rey Complex Figure Test, H = hemorrhagic stroke, I = ischemic stroke, MWT-B = Mehrfachwahl-Wortschatzintelligenztest, SIMD = substance-induced mood disorders, *SD* = standard deviation, TAP = Test Battery for Attention Performance, TAP-Alert = TAP-subtest “Alertness,” TAP-Go/NoGo = TAP-subtest “Go/NoGo,” TAP-VFCT = TAP-subtest “Visual field with central task,” WIE-BE = subtest “Bilder Ergänzen” (Picture Completion) from the German adaptation of the Wechsler Adult Intelligence Scale, *z* = *z*-scores. Statistical comparisons: *t* tests (with Welch correction in the presence of unequal variances). * Denotes significant results (*P* < 0.05). ^*a*^*z*-scores below -2 are listed as significant impairment, i.e., 2 *SD* below the mean of normative controls as provided by test manuals; ^*b*^indicative of mild depression.

All eight patients were evaluated on a range of standardized neuropsychological tests screening for cognitive, sensory, or language deficits. They also completed the Beck Depression Inventory-II (BDI-II; Beck et al., 1996), assessing depression symptoms at the time of testing. Comparisons between left- and right-injured patients on estimated IQ (Mehrfachwahl-Wortschatz-Intelligenztest [MWT-B]; Lehrl, 2005), perceptual reasoning (subtest “Bilder Ergänzen” [Picture Completion] from the German adaptation of the Wechsler Adult Intelligence Scale; Daseking and Petermann, 2013), indices of visuo-spatial attention (subtests “Alertness” and “Go/NoGo” from the Test Battery for Attention Performance [TAP]; Zimmermann and Fimm, 1997, respectively addressing phasic alertness and selective attention), or BDI-II revealed no significant group differences (all *P* ≥ 0.086, see Table 1). As shown in Table 1, visuo-spatial memory (memory quotient from the Rey Complex Figure Test, CFT-CQM; Meyers and Meyers, 1995) was significantly reduced in right-lesioned patients (*z*, 1.2 ± 0.5 [left] vs. 0.1 ± 0.7 [right], *t*_6_ = 2.642, *P* = 0.038). On single-subject level, however, patients did not score in the impaired range, i.e., >2 standard deviations (*SD*) below the mean of the normative control sample as provided by test manuals, except for patient R1 who was impaired in selective attention (*z_*R*__1_* = -2.2, Table 1). No patient exhibited BDI-II scores above the cut-off of 18 for clinically relevant depression (Hautzinger et al., 2006); the highest score of 16 (reached by patient R2) indicated mild depression according to common classification (Beck et al., 1996; Hautzinger et al., 2006). Visual field examination (TAP-subtest “Visual field with central task”) provided no evidence for manifest neglect (i.e., no blind spots). In addition to these examinations, all patients were asked to name two situations when people might feel disgusted, angry, happy, sad, or fearful in order to rule out potential deficits of conceptual emotional knowledge (Straube et al., 2010). No such deficits were found.

Nineteen male controls matched for age (54.7 ± 11.0 years [controls] vs. 58.8 ± 12.9 years [patients], *t*_25_ = 0.831, *P* = 0.411) and education level (16.9 ± 2.1 years [controls] vs. 16.5 ± 2.3 years [patients], *t*_25_ = 0.439, *P* = 0.664) were recruited via public announcements in local newspapers (*n* = 4), on university campus (*n* = 5), and in community groups (*n* = 10). All control participants reported no history of substance abuse, neurological, or psychiatric conditions. Patients and controls did not differ on BDI-II scores (5.5 ± 4.4 [controls] vs. 6.4 ± 5.4 [patients], *t*_25_ = 0.457, *P* = 0.652). All participants had normal or corrected-to-normal visual acuity and were right-handed and naïve to the study’s intent. Informed written consent was obtained from every participant, and financial compensation was given for participation. The experimental procedures conformed to the Declaration of Helsinki and were approved by and performed in accordance with the guidelines of the Ethical Commission of the Faculty of Social and Behavioral Sciences at the Friedrich Schiller University Jena (FSV 10/06).

### Lesion Reconstruction

Lesion location was confirmed by high resolution T_1_-weighted anatomical volumes (192 slices, TE = 5 ms, matrix = 256 mm × 256 mm, resolution = 1 mm × 1 mm × 1 mm; duration = 12 min) obtained with a 1.5 T magnetic resonance scanner (“Magnetom Vision Plus,” Siemens, Medical Systems, Erlangen, Germany). To quantify the volumetric extent of lesions, anatomical images were first aligned to Montreal Neurological Institute (MNI) space by applying the Clinical Toolbox for SPM12 (Wellcome Trust, London, United Kingdom), which includes a specialized standard MRI template and spatial normalization algorithms recommended for stroke-aged populations (Rorden et al., 2012). Clusterize Toolbox (version 1.0beta) available for SPM12 was applied for subsequent semi-automated lesion demarcation (de Haan et al., 2015). In an automated preprocessing procedure, each image was clustered based on local intensity maxima and iterative region adaptation. Minimum size for the initiation of a cluster was set to 150 mm^3^, avoiding oversegmentation. Lower intensity threshold of 20% was set, eliminating irrelevant background voxels, and iteratively adapted in steps of 1%, resulting in the assignment of each voxel to one cluster at each intensity threshold (de Haan et al., 2015). Clusters consistent with damaged tissue were then manually selected from the optimal intensity plane and, if necessary, amended by experts in clinical neuroimaging (CM, NM, and WS) who were blind to the study’s hypotheses, data, and statistical analyses at this time. MriCron^1^ was used to create within-group overlaps and map them on a standard brain. Standard anatomical atlases implemented in MRICron, i.e., Automated Anatomic Labeling (AAL) atlas and JHU white matter tractography atlas, were used to determine the extent of regional damage. A claustrum region of interest was added to the analyses, since claustrum is not part of the AAL atlas but was frequently affected in both patient groups due to its anatomical proximity to the IC and BG. Damage of IC-subregions was assessed using the parcellation from the Human Brainnetome Atlas^2^ (Fan et al., 2016).

### Emotional Test Battery

#### Test of Facial Expression Recognition

To assess the recognition and naming of basic emotions conveyed by facial expression, we employed an established paradigm based on morphing sequences of images from the standard Pictures of Facial Affect database (Young et al., 1997). Each morphing sequence contained 21 images of one model, each shown for 1,000 ms, which blended gradually from a 0% (neutral) to a 100% image (full-blown emotional expression) in 5% increments. The images contained frontal gray-scaled shots of faces that were size-adjusted, cropped to exclude non-facial clues, and presented in the center of a 27” computer screen on a black background. A blank black screen followed each morphing sequence for 1,000 ms, a fixation cross appeared for 500 ms, and a further blank screen shown for 1,000 ms introduced the next sequence. Morphing sequences were created for six emotional expressions (disgust, fear, sadness, anger, happiness, and surprise) in six models (three males, three females); each of these 36 sequences was presented two times in random order, with no repetition of sequences belonging to the same emotional category. Subjects were asked to watch the sequences carefully and to press a button as soon as the emotional content of the face was clear to them. After the button was pressed, a choice of six emotions appeared on the screen and remained until an answer was given. Two practice trials (one male, one female face) preceded the experimental trials, ensuring that every participant correctly understood the instructions. Accuracy of emotion recognition and the morphing level of reaction were measured and afterward multiplied (with morphing level inversed) to form a single index of recognition performance.

#### Test of Emotion Induction by Visual Scenes

The emotional induction task featured 60 standardized visual scenes from the International Affective Picture System (IAPS; Lang et al., 2008), aiming to induce basic emotional states of disgust, happiness, sadness, anger and fear, as well as a neutral state (no emotion), as proven by previous patient studies by our group (Straube et al., 2010; Holtmann et al., 2018). Each image was presented for 3,000 ms in the center of a 27” computer screen on a black background. Presentation was randomized, with no repetition of images aiming to induce the same target emotion. Subjects were instructed to watch the scenes carefully and to name their most prominent emotional sensation afterward by selecting one of six options. Response time was not limited, although subjects were asked to respond as fast as possible. Two scenes that were not included in the main test were used for practice purpose. The relative number of answers given in accordance with the target emotion provided an index of altered emotional functioning, as indicated by previous patient studies.

#### Affective Questionnaires

Two questionnaires targeting subjective report were administered after the previous experimental tasks were completed. In the first questionnaire, subjects rated how frequently and how intensely they experience the emotions disgust, fear, sadness, anger, and happiness in their daily lives on a 5-point scale. This questionnaire has been developed for investigating changes of emotional processing after brain injury (Berlin et al., 2004). In the second questionnaire, subjects rated how intensely they would experience disgust given 37 scenarios on a 5-point scale (Schienle et al., 2002). This Questionnaire for the Assessment of Disgust Sensitivity (QADS) is a frequent and valid measure in clinical literature on disgust processing.

### Statistical Analysis

Group differences on single emotional tests (dependent variables: accuracy and latency of emotion recognition from faces combined to a single index of emotion recognition, percentage of ratings given in accordance with the emotion targeted by emotional scenes, intensity and frequency of emotional experience, and sum score from QADS in case of disgust) were assessed by usage of contrasts that compared each of the patient groups with each other and with the healthy controls.

Previous evidence has suggested marked individual differences in disgust responsivity (Boucher et al., 2015; Tybur et al., 2018) and response inconsistency across single disgust domains (Woolley et al., 2015; Hall et al., 2016). In patient studies with relatively small sample sizes, this introduces the problem of individual single measures being valid indices only for an even smaller subgroup of patients. Therefore, we aggregated the different measures of each emotion into a single composite score per emotion in order to maximize psychometric reliability due to the degree of shared variance between the single measures (e.g., a common disgust factor), while at the same time maximizing the psychometric validity due to the degree of idiosyncratic variance (e.g., individual disgust facets). Analyses of internal consistency (see Supplementary Table 3) showed low inter-measure correlations, suggesting that each measure, although being an established measure of a feature of emotion processing, was only weakly representing a common factor. This might be related, at least partially, to true independence of different facets of emotion processing (Hall et al., 2016). This also means that single measures will not reliably indicate disgust deficits in patients with idiosyncratic lesions and/or disgust-related functional anatomy. In either case, a composite score would grasp the changes across emotional domains, thus yielding a better predictor of altered emotional processing than single constituent indicators (Carvalho and Vieta, 2017), as shown by studies assessing pain (O’Neill et al., 2014), emotional intelligence (Deater-Deckard et al., 2016; Hall et al., 2016; Zeigler-Hill and Shackelford, 2018), or cognitive abilities (Carvalho and Vieta, 2017). Emotion-specific composite scores were formed by (1) transforming each single test score into a *z*-standardized score and (2) averaging the *z*-standardized scores obtained for each emotion. Since disgust composites (but no other emotional composites) included a trait measure (QADS), we computed disgust scores discounting QADS data.

The composite scores showed a normal distribution (*P* ≥ 0.127). Group comparisons were performed for composite scores of each emotional category, using one-way ANOVAs. Significant group effects were further investigated via two-sided two-sample *t*-tests (LSD corrected). Complementary analyses in form of two-sided two-sample Bayesian *t-*tests (with a default Cauchy prior width of *r* = 0.707) were added to secure findings of significant differences between patient groups on emotion-specific composite scores. Since right-lesioned patients exhibited somewhat higher total (Table 1) and BG lesion volume (Supplementary Table 1), and scored significantly lower on visuo-spatial memory (Table 1), analyses of covariance (ANCOVAs) were added, with total lesion volume, BG lesion volume, and visuo-spatial memory scores as respective covariates to exclude the possibility that a difference between the patient groups on emotion-specific composite scores was attributable to differences in neurological and cognitive characteristics. Differences were considered significant at *P* < 0.050. Analyses were performed using IBM SPSS Statistics for Windows, Version 25 (IBM Corp. Armonk, NY, United States) and JASP, Version 0.8.6 (Jasp Team, Amsterdam, Netherlands).

## Results

### Single Measure Data

Table 2 lists summary data by group and emotion, as well as group difference *P*-values in the separate tasks. Task performance across patients and controls was heterogeneous (see Supplementary Table 4 listing individual test scores for disgust). Although disgust-related means of the left-lesioned group were lower than those of the right-lesioned group across all tests, no significant differences between the patient groups were found (all *P* ≥ 0.069). Significant group differences emerged for the intensity of disgust experience in the daily life, with left-lesioned patients scoring lower than controls (*t*_21_ = 2.776, *P* = 0.011), and the QADS, with right-lesioned patients scoring higher than controls (*t*_21_ = 2.376, *P* = 0.027). Regarding other emotional conditions, significant group differences were found for the intensity of everyday experience of happiness, with right-lesioned patients scoring higher than controls (*t*_18_ = 0.2.348, *P* = 0.031), and the experience of sadness toward sad scenes, with left-lesioned patients scoring lower than controls (*t*_7_._59_ = 2.915, *P* = 0.021).

**TABLE 2:** Emotion-specific test performance scores and composite scores in patients and controls.

		Left-lesioned patients *M* ± *SE*	Control group *M* ± *SE*	Right-lesioned patients *M* ± *SE*	Left-lesioned patients vs. controls *P*	Right-lesioned patients vs. controls *P*	Left vs. right-lesioned patients *P*
Disgust	Faces: accuracy x latency [0; 1]	0.18 ± 0.08	0.21 ± 0.03	0.27 ± 0.05	0.718	0.363	0.347
	Scenes: percentage match (%)	0.75 ± 0.13	0.83 ± 0.04	0.98 ± 0.03	0.469	0.153	0.187
	Frequency [1;5]	1.50 ± 0.29	2.11 ± 0.21	2.25 ± 0.48	0.232	0.782	0.228
	Intensity [1;5]	1.25 ± 0.25	2.47 ± 0.19	2.75 ± 0.63	0.011*	0.588	0.069
	QADS [1; 5]	2.40 ± 0.30	2.23 ± 0.15	3.19 ± 0.53	0.648	0.027*	0.253
	Composite (*z*)	−0.49 ± 0.22	−0.02 ± 0.09	0.57 ± 0.14	0.029*	0.008*	< 0.001*
Happiness	Faces: accuracy x latency [0; 1]	0.40 ± 0.17	0.50 ± 0.03	0.44 ± 0.07	0.611	0.446	0.861
	Scenes: percentage match (%)	0.88 ± 0.06	0.83 ± 0.05	0.95 ± 0.03	0.660	0.258	0.320
	Frequency [1; 5]	3.75 ± 0.25	3.53 ± 0.19	3.25 ± 0.25	0.617	0.538	0.207
	Intensity [1;5]	3.50 ± 0.29	3.63 ± 0.16	4.00 ± 0.00	0.725	0.031*	0.134
	Composite (*z*)	−0.06 ± 0.25	−0.01 ± 0.16	0.12 ± 0.08	0.896	0.454	0.514
Sadness	Faces: accuracy x latency [0; 1]	0.06 ± 0.04	0.20 ± 0.04	0.12 ± 0.03	0.105	0.381	0.195
	Scenes: percentage match (%)	0.70 ± 0.04	0.85 ± 0.03	0.73 ± 0.05	0.021*	0.105	0.705
	Frequency [1;5]	2.50 ± 0.29	2.58 ± 0.23	2.50 ± 0.65	0.883	0.894	0.999
	Intensity [1;5]	3.00 ± 0.71	2.95 ± 0.19	2.75 ± 0.63	0.921	0.700	0.801
	Composite (*z*)	−0.38 ± 0.23	0.14 ± 0.11	−0.28 ± 0.38	0.071	0.172	0.843
Fear	Faces: accuracy x latency [0; 1]	0.11 ± 0.06	0.18 ± 0.03	0.20 ± 0.07	0.270	0.759	0.331
	Scenes: percentage match (%)	0.58 ± 0.11	0.75 ± 0.06	0.58 ± 0.15	0.214	0.237	0.999
	Frequency [1;5]	2.25 ± 0.48	2.00 ± 0.22	2.00 ± 0.41	0.636	0.999	0.705
	Intensity [1;5]	2.50 ± 0.65	2.37 ± 0.21	1.75 ± 0.25	0.806	0.200	0.320
	Composite (*z*)	−0.14 ± 0.27	0.08 ± 0.12	−0.22 ± 0.34	0.472	0.350	0.865
Anger	Faces: accuracy x latency [0; 1]	0.16 ± 0.09	0.18 ± 0.03	0.15 ± 0.07	0.478	0.400	0.958
	Scenes: percentage match (%)	0.78 ± 0.03	0.76 ± 0.04	0.58 ± 0.18	0.875	0.371	0.337
	Frequency [1;5]	3.75 ± 0.48	2.63 ± 0.23	2.75 ± 0.48	0.056	0.832	0.190
	Intensity [1;5]	4.00 ± 0.41	3.21 ± 0.21	3.75 ± 0.48	0.127	0.300	0.705
	Composite (*z*)	0.36 ± 0.12	−0.03 ± 0.11	−0.2 ± 0.38	0.138	0.579	0.206

QADS: Questionnaire for the Assessment of Disgust Sensitivity; * *P* < 0.050, uncorrected (*LSD*-corrected for disgust composites), statistics: *t* tests.

### Composite Scores

**FIGURE 2:**
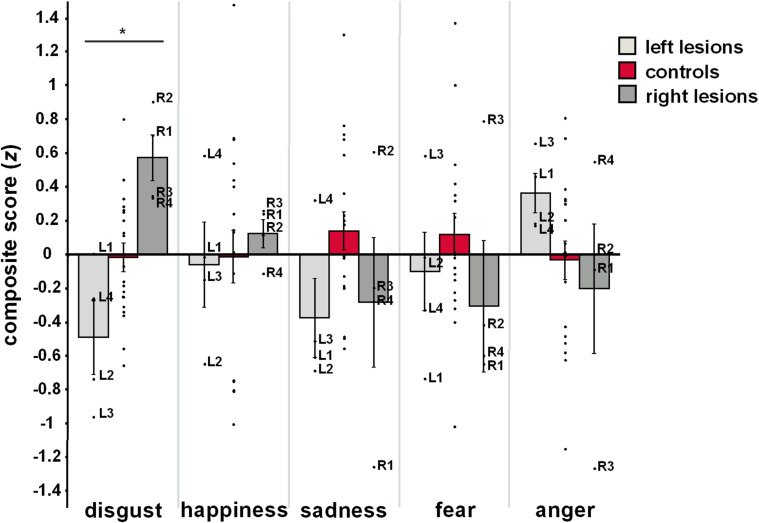
Mean and individual emotion-specific composite scores for each group. Error bars represent standard error of the mean. *P < 0.05, one-way ANOVA for main group effects. Left-lesioned patients: L1-4, right-lesioned patients: R1-4.

Figure 2 illustrates mean as well as individual composite scores from each group and for each emotional category. Analyses of group effects on emotion-specific composite scores revealed significant group differences for disgust (*F*_2_,_26_ = 8.26, *P* = 0.002), but not for other emotional categories (happiness: *F*_2_,_26_ = 0.10, *P* = 0.904; sadness: *F*_2_,_26_ = 2.20, *P* = 0.132; fear: *F*_2_,_26_ = 0.61, *P* = 0.551; anger: *F*_2_,_26_ = 1.35, *P* = 0.278). *Post hoc* comparisons revealed that composite scores of disgust were reduced in patients with left-hemispheric lesions (*M* ± *SE*: –0.49 ± 0.22; *P* = 0.029) but heightened in patients with right-hemispheric lesions (0.57 ± 0.17, *P* = 0.008), when related to HC (–0.02 ± 0.09). Most importantly, the two patient groups exhibited significantly diverging patterns of disgust processing (*P* < 0.001). This effect did not change when QADS was removed from the computation of the disgust composite score to account for the lack of trait measures for other basic emotional categories (left-lesioned patients: -0.61 ± 0.20 vs. right-lesioned patients: 0.46 ± 0.22, *P* = 0.001).

### Complementary Analyses

Bayesian hypothesis testing yielded a Bayes factor of BF10 = 7.00, indicating that, given the observed data, the alternative hypothesis (i.e., that left-lesioned patients showed different disgust composite scores than right-lesioned patients) was seven times more likely than the null hypothesis. This result pleads for substantial evidence according to common classifications.

In the ANCOVA models, separately adjusting for overall lesion volume, BG lesion volume, and visuo-spatial memory, the difference between left- and right-lesioned patients on the disgust composite score remained (total lesion volume: -0.5 ± 0.2 [left] vs. 0.6 ± 0.2 [right], *F*_1_,_5_ = 9.588, *P* = 0.027; basal ganglia volume: -0.3 ± 0.1 [left] vs. 0.4 ± 0.1 [right], *F*_1_,_5_ = 13.490, *P* = 0.019; visuo-spatial memory: -0.4 ± 0.2 [left] vs. 0.5 ± 0.2 [right], *F*_1_,_5_ = 4.785, *P* = 0.040, one-sided).

## Discussion

The present study systematically examined effects of lateralized IC-BG lesions on disgust processing. Using well-established emotional measures, we compared profiles of basic emotion processing in patients with left or right IC-BG damage and controls. No single tests provided a statistically clear-cut separation of the patient groups. However, aggregation of different single test scores into one emotion-specific composite score indicated generalized changes for disgust, but not for other emotions. Left-lesioned patients exhibited significantly lower disgust composite scores than right-lesioned patients, or healthy controls. Right-lesioned patients showed significantly higher disgust composites than controls. The study supports the central role of the IC-BG complex in disgust. Crucially, it argues for a primarily left-hemispheric basis of disgust.

The role of the IC-BG complex in disgust has been controversial due to inconsistent findings in the human lesion literature. Disproportionate disgust impairments were shown in single patients with left (Calder et al., 2000; Borg et al., 2013) or bilateral (Adolphs et al., 2003) IC-BG damage. No superior role of the IC-BG in disgust was shown in single right-lesioned patients (Straube et al., 2010; Couto et al., 2013; Terasawa et al., 2015). Our findings provide an explanatory basis for these disparate findings, as our well-controlled comparisons of patient groups with lateralized IC-BG damage imply idiosyncratic involvement of the right and left IC-BG complex in disgust. We found that left, but not right, IC-BG damage resulted in a substantial decrease of disgust processing. Thus, in line with previous single-case studies, disgust deficits may draw on left rather than right IC-BG damage, suggesting the left IC-BG as the functional basis of disgust. This conclusion is consistent with the larger number of functional neuroimaging studies supporting left rather than right IC activation to disgust stimuli (Sprengelmeyer et al., 1998; Zald and Pardo, 2000; Royet et al., 2003; Small et al., 2003; Wicker et al., 2003). Further support comes from recent electrostimulation studies in humans (Papagno et al., 2016) and monkeys (Caruana et al., 2011), and from voxel-based morphometry studies in neurodegenerative samples (Kipps et al., 2007; Kumfor et al., 2013) that demonstrate the direct role of the left IC in disgust, although these studies did not explicitly test for differential hemispheric involvement.

What may be the potential psychobiological mechanism driving this lateralized functional difference? Physiological states are considered the basis for emotion and feeling (Damasio, 1994; Craig, 2002). The left IC supports the modulation of the parasympathetic tone (Oppenheimer et al., 1996; Craig, 2005; Guo et al., 2016), the right IC the regulation of the sympathetic tone (Oppenheimer et al., 1992; Craig, 2005; Beissner et al., 2013). Cortical representation, control, and regulation of autonomic tone are fundamentally organized by coordinated opponent inhibition (Craig, 2005). Lateralized autonomic input to the IC may drive lateralized processing of emotions, relying on either sympathetic or parasympathetic activation (Craig, 2005; Duerden et al., 2013). Activation of the parasympathetic nervous system is essential in initiating disgust responses (Levenson, 2014). Experimental deactivations or stroke affecting the left IC have been linked with increased sympathetic and correspondingly decreased parasympathetic tone (Oppenheimer et al., 1996; Hilz et al., 2001). Thus, left IC lesions may disinhibit the right hemisphere, exaggerating the sympathetic tone and, by disrupting an area of parasympathetic representation, potentially distorting integration of information relevant to correctly process disgust. This may explain decreased disgust processing after left IC-BG lesions in the present study. Complementarily, a recent study of patients with frontotemporal dementia, in whom decremented disgust responding is known to depend on bilateral (Woolley et al., 2015) and left IC atrophy (Kumfor et al., 2013), provided evidence that left IC degeneration is associated with parasympathetic dysfunction (Guo et al., 2016). In contrast, increased parasympathetic tone has been noticed after right IC lesions (Oppenheimer et al., 1992; Nagai et al., 2016). Thus, right forebrain lesions may result in left-sided excitation due to lacking control of the left forebrain, thereby enhancing the presentation of parasympathetic activation. This may preserve or even boost disgust responses, as observed in the present study. Taken together, left-lateralized disgust processing may be due to left-focussed parasympathetic representation. The specific pattern of altered disgust processing after left resp. right IC-BG lesions may be rooted in abrogated interhemispheric opponent regulation. Further research is needed to explicitly address the link between lateralization of disgust and differential patterns of autonomic activity in lesion models of lateralized damage.

Our results support and refine the disproportionate role of the IC-BG complex in disgust (Krolak-Salmon et al., 2003; Fusar-Poli et al., 2009; Chapman and Anderson, 2012; Lindquist et al., 2012; Vicario et al., 2017) and may contribute to the assessment and treatment of neuropsychiatric disorders that, in part, are characterized by IC-BG dysfunction (for review see Vicario et al., 2017). However, there is evidence suggesting IC-BG involvement in emotion-general functions (Wager et al., 2003, 2015). These functions may explain some broad emotional deficits observed after IC-BG damage (Borg et al., 2013; Couto et al., 2013; Terasawa et al., 2015; Vicario et al., 2017). The IC is critically involved in interoception and salience detection (Menon and Uddin, 2010; Namkung et al., 2017). Fomenting orientation responses to imminent visceral or somatic experiences, IC supports the prioritized processing of interoceptive events with consequences for bodily homeostasis (Wager et al., 2015; Woolley et al., 2015). More than other emotional constructs, disgust is proposed to capitalize on these IC functions, being the most visceral of all basic emotions (Chapman and Anderson, 2012; Woolley et al., 2015; Verstaen et al., 2016). Our results resonate with this position. Besides large disgust-related effects, both patient groups exhibited reduced composites for fear and sadness. No group differences occurred in our standard ANOVA. When combining the patient groups, a significant difference between the patient and the control group would emerge for sadness (*P* = 0.044) but not fear (*P* = 0.278). Thus, IC-BG lesions may reduce sadness. Sadness measures are related to empathy for others. The IC is crucially involved in empathic processes triggered by the perception of others’ emotions and understanding of others’ emotions (Singer et al., 2009; Engen and Singer, 2013). Future studies should address the relation between IC lesions, experimentally induced sadness, and empathy for others.

In line with previous research (Boucher et al., 2015; Woolley et al., 2015; Vicario et al., 2017), single-test performance was heterogeneous and individual differences in test performance produced overlaps that aggravated clear-cut separations of groups, leaving it unclear on which domains to study functional lateralization (Duerden et al., 2013; Kann et al., 2016). Composite indicators may be more robust indicators of emotional processing than single tests. First, emotions are commonly viewed as composite and multi-component constructs (Adolphs, 2017; Celeghin et al., 2017). Therefore, we conflated information from well-established, face-valid, and conceptually meaningful indicators of different emotional components. Accordingly, analyses of internal consistency revealed variable inter-indicator correlations, meaning that some indicators drew on the same underlying component, and others not. The advantage of conflating such indicators is the maximization of information contributed to the overall concept, thus avoiding a test battery being redundant and too narrow in scope to represent adequately the dispositional variable (Epstein, 1983; Markus and Borsboom, 2013). Second, single items of behavior are inconsistent, may be poorly correlated, and are limited to predict broad response dispositions (Diener and Larsen, 1984; Hall et al., 2016; Zeigler-Hill and Shackelford, 2018). Stable dispositional patterns may emerge when behavior is aggregated across stimuli and situations (Epstein, 1983; Diener and Larsen, 1984), capturing the depth and breadth of individual differences (Deater-Deckard et al., 2016). However, we are aware that although we show selective changes for disgust after IC-BG damage at the composite-score level, this does not preclude other emotional changes in single patients. Future studies with increased sample size are needed to better understand individual differences in single-test response patterns of patients with left and right-hemispheric IC-BG damage for disgust and other basic emotions.

Studies with neuropsychiatric samples with IC-BG dysfunctions have shown detrimental effects of cognitive impairment, degree of volume loss, and task difficulty on the performance on emotional tasks (Suzuki et al., 2006; Gray and Tickle-Degnen, 2010; Argaud et al., 2018). In our study, right-lesioned patients exhibited somewhat higher total and BG lesion volume, and scored significantly lower on visuo-spatial memory than left-lesioned patients. However, right-lesioned patients performed significantly better for disgust than left-lesioned patients, or controls. They also responded differently across emotional categories. Moreover, individual cognitive performance was not in the impaired range (>2 SD below the mean of normative control samples), except for R1 (abnormal on selective attention, see Table 1). The difference between left and right patients on disgust remained, when visuo-spatial memory, total, and BG lesion volume were entered as covariates. Thus, cognitive performance and lesion size may not explain the better disgust performance in right-lesioned patients.

There are limitations to be noted. The major caveat is the small number of patients, a limitation imposed by the rarity of patients with circumscribed IC-BG lesions. Thus, we were able to detect large emotion effects, as for disgust, but given the general role of the IC-BG system in emotion, other emotional deficits might not have been unveiled. Second, although we show the usability of composite indicators for dissociating emotion processing after left vs. right IC-BG damage, we are aware that our findings are only preliminary and require validation. Our emotional test battery may need further refinement (e.g., by adding trait measures for other basic emotions), and the combination of indicators chosen for the composite score may be relevant. Third, future studies should address potential gender differences, since our inferences are based on results from male participants only. Gender differences have been observed for neural responses to emotional stimuli (Wager et al., 2003), specifically for the lateralization of emotional processing within the IC (Duerden et al., 2013). Finally, future research should address effects of lesion age on the present results.

We investigated patients with extended lesion areas, which aggravates definite inferences on the neural underpinnings of disgust. The IC is most reliably linked to disgust. Yet, this role may be imparted from its interconnections with the BG (Lewis et al., 2008). While the IC subserves the detection of disgust, the BG support the recognition of disgust, withdrawal motivation, the selection and initiation of appropriate escape, or defensive behavior (Royet et al., 2003; Lewis et al., 2008; Obeso et al., 2014; Eisinger et al., 2018). The ventral striatum (nucleus accumbens), a core hub in the reward circuit, supports the recognition and anticipation of disgust, aiding translation to motivated behavior (Heining et al., 2003; Arias-Carrión et al., 2010; Eisinger et al., 2018). The ventral pallidum, a “hedonic hot spot,” supports the sensory experience of disgust, thus controlling negative motivation to produce avoidance behavior (Calder et al., 2007; Smith et al., 2009; Ho and Berridge, 2014). The dorsal striatum (putamen, caudate) is involved in goal-directed behavior (i.e., action selection and initiation, inhibitory control, reappraisal of disgust) through the integration of sensorimotor, cognitive, and motivational information (Balleine et al., 2007; Gonçalves et al., 2016; Eisinger et al., 2018). Note that Boucher et al. (2015) reported rather subtle emotional deficits in post-surgery epileptic patients with unilateral operculo-insular resections. Although this null finding may reflect seizure-induced synaptic reorganization due to a long history of epileptic seizures (3–34 years) in these patients, it supports the view that not IC injury *per se*, but rather the disruption of the insular-striatal-pallidal-thalamic-insular circuit may have determined the present findings, particularly the disgust deficit in the left-lesioned group. Yet, Boucher et al. (2015) studied specific emotional changes irrespective of lesion laterality, and the higher proportion of right-lesioned IC patients (*n*_right_ = 9 vs. *n*_left_ = 6) may have resulted in (averagely) better recognition of disgust faces in the IC group compared to the healthy and lesion-control group. The authors explored lateralization effects on mean performance across all emotional conditions, showing somewhat greater global emotional decrements after left IC injury. The results from our study, however, highlight the necessity to test for lateralization effects emotion-specifically, because lateralization might be an essential aspect in the interpretation of functions attributed to the IC and of its role in cortico-subcortical networks forming the neural basis of emotion. Future studies need to refine distinct neural contribution to disgust. Our results suggest hemispheric lateralization as an expedient basis for the investigation of functional involvement of single nodes and their connections within and between hemispheres, for example by applying dynamic causal modeling. Here, effects of IC subregions on disgust deserve particular interest (Craig, 2002; Vicario et al., 2017).

In conclusion, upon comparisons of patients with lateralized IC-BG damage, we argue for a distinct hemispheric involvement in disgust. Based on data aggregation, we suggest a left-hemispheric basis of disgust processing, potentially due to asymmetrical representations of peripheral autonomic nervous activity in the human forebrain.

## Data Availability Statement

The datasets generated during this study are partially included in this article/Supplementary Material or are available from the corresponding author on reasonable request.

## Ethics Statement

The studies involving human participants were reviewed and approved by Ethical Commission of the Faculty of Social and Behavioral Sciences at the Friedrich Schiller University Jena (FSV 10/06). The patients/participants provided their written informed consent to participate in this study.

## Author Contributions

TS and WM designed the study. OH conducted the data collection, the statistical analyses with the support of MB, the lesion analyses with the support of CM and MB, and wrote the first draft of the article. WS and NM provided expert advice on lesion location. All authors contributed to revising the final version of the manuscript.

## Conflict of Interest

The authors declare that the research was conducted in the absence of any commercial or financial relationships that could be construed as a potential conflict of interest.

## Footnotes

1 http://www.mccauslandcenter.sc.edu/crnl/tools

2 http://atlas.brainnetome.org
