# Supplementary material for: Lateralized Deficits of Disgust Processing After Insula-Basal Ganglia Damage
Source: Front Psychol. 2020 Jun 30;11:1429. doi: 10.3389/fpsyg.2020.01429 (PMC7347022; doi:10.3389/fpsyg.2020.01429)
Supplement: Supplementary file 1 [file Data_Sheet_1.docx]

Supplementary Material

**
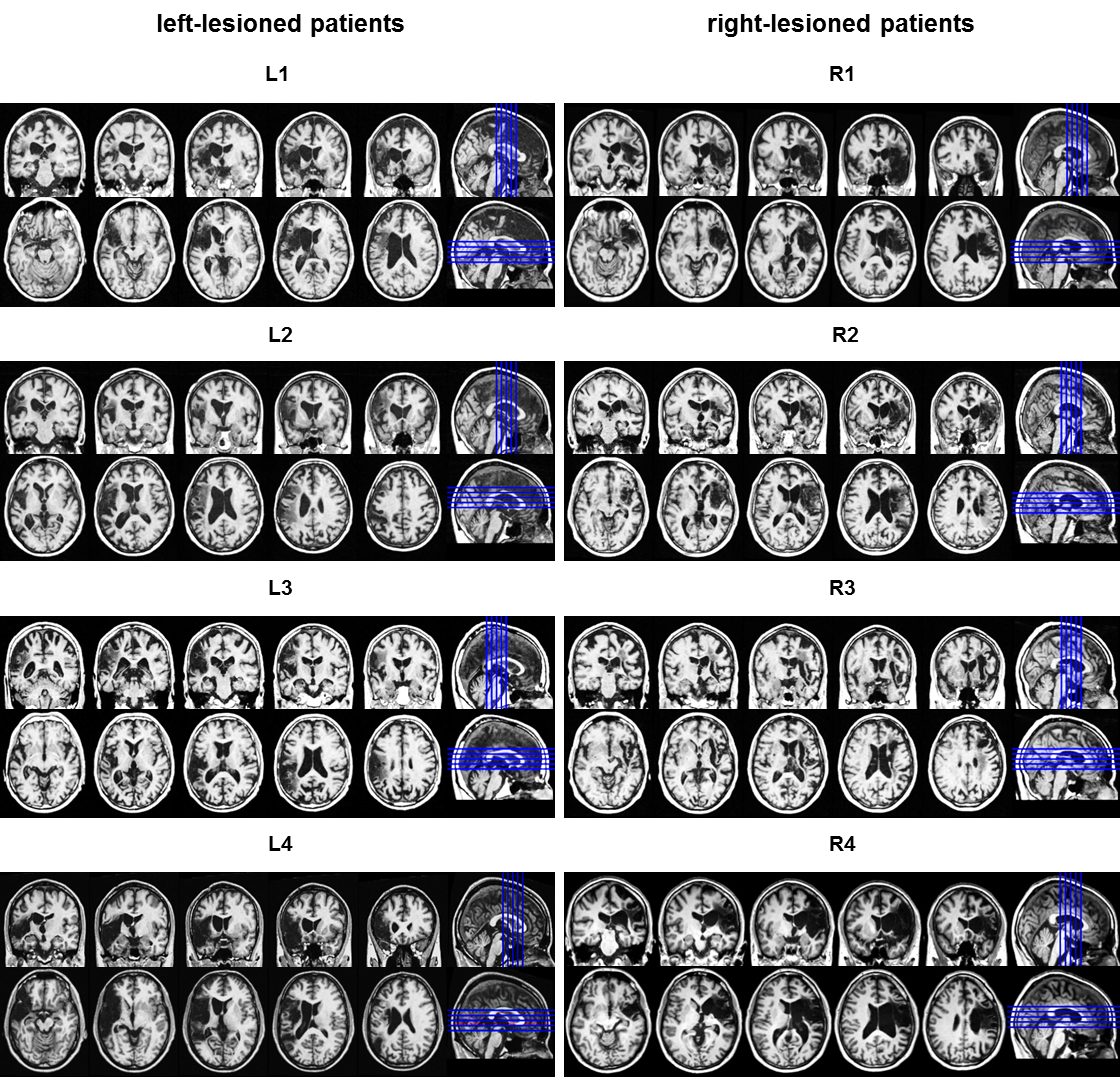
**

**Supplementary Figure 1:** MNI-normalized T1-weighted coronal and axial MRI scans from individual patients. **L1:** ischemic stroke, mainly affecting the insula (completely), caudate nucleus, claustrum, putamen, globus pallidus, inferior frontal gyrus, rolandic operculum, medial temporal lobe, internal, extreme and external capsule, superior fronto-occipital fasciculus, fasciculus uncinatus, parts of corona radiata and tapetum. **L2:** ischemic stroke, with main damage to the insula (complete), claustrum, inferior frontal gyrus, rolandic operculum, postcentral gyrus, inferior parietal gyrus, supramarginal gyrus, transverse and superior temporal gyrus, parts of internal capsule and corona radiata, extreme capsule, as well as superior longitudinal and fronto-occipital fasciculus. **L3:** ischemic stroke, with main damage to the posterior insula, claustrum, inferior frontal gyrus, postcentral gyrus, rolandic operculum, supramarginal gyrus, transverse, superior and middle temporal gyrus, external and extreme capsule, as well as superior longitudinal fasciculus. **L4:** ischemic stroke with main damage given in the insula (completely lost), caudate nucleus, claustrum, putamen, inferior frontal gyrus, rolandic operculum, transverse and superior temporal gyrus, temporal pole, internal, extreme and external capsule, as well as fasciculus uncinatus and superior fronto-occipital fasciculus. **R1:** ischemic stroke, primarily affecting the insula (entirely), caudate nucleus, claustrum, putamen, globus pallidus, inferior frontal gyrus, rolandic operculum, transverse and superior temporal gyrus, temporal pole, internal, extreme and external capsule, corona radiata, fasciculus uncinatus, superior longitudinal and fronto-occipital fasciculus. **R2:** ischemic stroke, primarily affecting the insula (entirely), caudate nucleus, claustrum, putamen, globus pallidus, inferior frontal gyrus, rolandic operculum, transverse and superior temporal gyrus, temporal pole, internal, extreme and external capsule, corona radiata, fasciculus uncinatus, superior longitudinal and fronto-occipital fasciculus. **R3:** hemorrhagic stroke, predominantly affecting the posterior ventral insula, claustrum, putamen, globus pallidus (lateralis), inferior and medial frontal gyrus, transverse temporal gyrus, temporal pole, internal, extreme and external capsule, parts of corona radiate, superior longitudinal and fronto-occipital fasciculus, as well as fasciculus uncinatus. **R4:** ischemic stroke, predominantly affecting the insula (completely), caudate nucleus, claustrum, putamen, globus pallidus, pre- and postcentral gyrus, inferior frontal gyrus, rolandic operculum, supramarginal gyrus, thalamus, transverse and superior temporal gyrus, temporal pole, internal, extreme and external capsule, corona radiata, superior longitudinal as well as fronto-occipital fasciculus, and fasciculus uncinatus. Lesion location was determined by means of standard anatomical atlases in MRIcron (AAL atlas, JHU white matter atlas).

**Supplementary Table 1: Comparative overview of regional lesion volume**

|  | region |  | right lesions  *M* ± *SE* |  | left lesions  *M* ± *SE* |  | *P* |
| --- | --- | --- | --- | --- | --- | --- | --- |
| insula | |  | 12.89 ± 0.75 |  | 10.29 ± 1.99 |  | 0.291 |
| basal ganglia | |  | 15.62 ± 2.24 |  | 8.23 ± 3.77 |  | 0.143 |
| white matter tracts adjacent to insula and basal ganglia | |  | 13.26 ± 1.19 |  | 7.06 ± 2.94 |  | 0.123 |
| thalamus | |  | 3.97 ± 0.68 |  | 2.72 ± 1.03 |  | 0.348 |
| limbic structures | |  | 3.83 ± 0.47 |  | 2.16 ± 0.96 |  | 0.167 |
| temporal lobe | |  | 36.54 ± 3.15 |  | 34.20 ± 10.41 |  | 0.837 |
| central regions | |  | 18.53 ± 9.82 |  | 17.17 ± 4.84 |  | 0.905 |
| frontal lobe | |  | 25.67 ± 5.73 |  | 17.87 ± 4.77 |  | 0.336 |
| parietal lobe | |  | 7.64 ± 4.22 |  | 13.28 ± 7.25 |  | 0.532 |
| occipital lobe | |  | 0.56 ± 0.32 |  | 1.55 ± 0.38 |  | 0.092 |
| white matter tracts | |  | 15.07 ± 0.27 |  | 10.5 ± 1.38 |  | **0.043** |
| unclassified tissue | |  | 24.64 ± 6.82 |  | 16 ± 1.62 |  | 0.263 |

For each anatomical subdivision, lesion volume is reported in cm³. Standard atlases (AAL atlas, JHU white matter atlas) were used to determine lesion localization and lesion volume on T_1_-weighted MR images normalized to MNI space. A claustrum region of interest was added to the analyses, since claustrum is not included in the AAL atlas. Basal ganglia damage covered damage to the caudate nucleus, putamen, pallidum and claustrum. Damage of limbic structures comprised damage to the hippocampus, amygdala, cingulate gyrus and parahippocampal gyrus. Temporal lobe damage referred to damage to the temporal pole, superior, transverse, middle and inferior temporal gyrus. Analyses of damage to central regions, frontal, parietal and occipital lobes followed the anatomical parcellation proposed by Rolls and colleagues (2015). Damage to external and internal capsule, anterior corona radiata and uncinate fasciculus was subsumed under damage to white matter tracts adjacent to insula and basal ganglia. Damage to all other deep white matter structures included in the JHU white matter atlas was subsumed under damage to white matter tracts. Unclassified tissue refers to damaged regions that were not included in the atlases. Significant results (P < .05, uncorrected for multiple comparisons) are printed in bold. Statistical comparisons: t tests (with Welch correction in the presence of unequal variances).

Rolls, E. T., Joliot, M., and Tzourio-Mazoyer, N. (2015). Implementation of a new parcellation of the orbitofrontal cortex in the automated anatomical labeling atlas. *NeuroImage* 122, 1–5. doi:10.1016/j.neuroimage.2015.07.075.

**Supplementary Table 2: Comparative overview of regional IC lesion volume**

| IC subregion |  | right lesions  *M* ± *SE* |  | left lesions  *M* ± *SE* |  | *P* |
| --- | --- | --- | --- | --- | --- | --- |
| hypergranular |  | 0.8 ± 0.04 |  | 0.7 ± 0.09 |  | 0.283 |
| ventral agranular |  | 0.9 ± 0.05 |  | 0.5 ± 0.3 |  | 0.188 |
| dorsal agranular |  | 0.9 ± 0.03 |  | 0.6 ± 0.2 |  | 0.210 |
| ventral dysgranular/ granular |  | 1.0 ± 0.01 |  | 0.7 ± 0.1 |  | 0.133 |
| dorsal granular |  | 0.9 ± 0.07 |  | 0.9 ± 0.05 |  | 0.977 |
| dorsal dysgranular |  | 0.9 ± 0.06 |  | 0.9 ± 0.1 |  | 0.598 |

Note: For each subregion, the extent of damage is reported in %. Analysis of regional IC damage was based on the Human Brainnetome Atlas (http://atlas.brainnetome.org; Fan et al., 2016). Statistical comparisons: t tests (with Welch correction in the presence of unequal variances). *P*-values are uncorrected for multiple comparisons.

Fan, L., Li, H., Zhuo, J., Zhang, Y., Wang, J., Chen, L., et al. (2016). The Human Brainnetome Atlas: A New Brain Atlas Based on Connectional Architecture. *Cereb. Cortex N. Y. N 1991* 26, 3508–3526. doi:10.1093/cercor/bhw157.

**Supplementary Table 3: Results from analyses of internal consistency (inter-test correlations and Cronbach’s alpha) for each emotion category**

|  |  | QADS | scenes | frequency | intensity | faces | α |
| --- | --- | --- | --- | --- | --- | --- | --- |
| disgust | QADS | 1.00 |  |  |  |  | 0.085 |
|  | scenes | 0.144 | 1.00 |  |  |  |  |
|  | frequency | 0.048 | -0.298 | 1.00 |  |  |  |
|  | intensity | -0.123 | -0.035 | -0.084 | 1.00 |  |  |
|  | faces | 0.251 | -0.088 | 0.431 | -0.064 | 1.00 |  |
| happiness | scenes |  | 1.00 |  |  |  | 0.414 |
|  | frequency |  | 0.020 | 1.00 |  |  |  |
|  | intensity |  | 0.325 | 0.549 | 1.00 |  |  |
|  | faces |  | -0.072 | -0.020 | 0.100 | 1.00 |  |
| sadness | scenes |  | 1.00 |  |  |  | 0.258 |
|  | frequency |  | -0.240 | 1.00 |  |  |  |
|  | intensity |  | -0.050 | 0.376 | 1.00 |  |  |
|  | faces |  | -0.058 | 0.299 | 0.154 | 1.00 |  |
| fear | scenes |  | 1.00 |  |  |  | 0.234 |
|  | frequency |  | -0.065 | 1.00 |  |  |  |
|  | intensity |  | 0.128 | 0.691 | 1.00 |  |  |
|  | faces |  | -0.176 | 0.102 | -0.255 | 1.00 |  |
| anger | scenes |  | 1.00 |  |  |  | 0.114 |
|  | frequency |  | -0.069 | 1.00 |  |  |  |
|  | intensity |  | -0.233 | 0.678 | 1.00 |  |  |
|  | faces |  | 0.205 | -0.230 | -0.163 | 1.00 |  |

*Note:* QADS: Questionnaire for the Assessment of Disgust Sensitivity. α: Cronbach’s α based on standardized items

**Supplementary Table 4: Individual disgust-related data on emotion-specific measures in patients and controls**

| patient | faces  accuracy x latency [0; 1] | scenes  percentage match (%) | frequency  [1;5] | intensity  [1;5] | QADS  [1; 5] |
| --- | --- | --- | --- | --- | --- |
| R1 | 0.31 | 1.00 | 3 | 3 | 2.46 |
| R2 | 0.14 | 0.90 | 3 | 4 | 3.95 |
| R3 | 0.34 | 1.00 | 1 | 1 | 4.22 |
| R4 | 0.31 | 1.00 | 2 | 3 | 2.11 |
| L1 | 0.36 | 0.90 | 1 | 1 | 3.27 |
| L2 | 0.23 | 0.70 | 1 | 1 | 1.97 |
| L3 | 0.00 | 0.40 | 2 | 2 | 2.08 |
| L4 | 0.15 | 1.00 | 2 | 1 | 2.27 |
| HC1 | 0.13 | 0.70 | 4 | 1 | 1.92 |
| HC2 | 0.11 | 1.00 | 2 | 3 | 2.92 |
| HC3 | 0.24 | 1.00 | 2 | 2 | 2.68 |
| HC4 | 0.24 | 0.60 | 3 | 3 | 2.95 |
| HC5 | 0.33 | 0.60 | 2 | 3 | 2.08 |
| HC6 | 0.38 | 0.80 | 2 | 2 | 2.84 |
| HC7 | 0.27 | 0.90 | 2 | 2 | 3.41 |
| HC8 | 0.35 | 0.80 | 2 | 4 | 2.11 |
| HC9 | 0.05 | 0.90 | 2 | 2 | 2.30 |
| HC10 | 0.02 | 1.00 | 1 | 2 | 1.57 |
| HC11 | 0.03 | 0.80 | 3 | 3 | 1.51 |
| HC12 | 0.34 | 1.00 | 2 | 2 | 1.73 |
| HC13 | 0.50 | 0.30 | 2 | 2 | 1.84 |
| HC14 | 0.18 | 0.70 | 3 | 3 | 2.32 |
| HC15 | 0.10 | 0.90 | 1 | 2 | 2.62 |
| HC16 | 0.24 | 0.80 | 4 | 4 | 2.49 |
| HC17 | 0.26 | 1.00 | 1 | 1 | 2.84 |
| HC18 | 0.26 | 1.00 | 1 | 3 | 1.57 |
| HC19 | 0.10 | 1.00 | 1 | 3 | 0.70 |

*Note:* HC = healthy controls, R = right-lesioned patients, L = left-lesioned patients, QADS: Questionnaire for the Assessment of Disgust Sensitivity.
